# Cell guidance ligands, receptors and complexes – orchestrating signalling in time and space

**DOI:** 10.1016/j.sbi.2019.11.007

**Published:** 2020-04

**Authors:** Daniel Rozbesky, Edith Yvonne Jones

**Affiliations:** Division of Structural Biology, Wellcome Centre for Human Genetics, University of Oxford, Oxford, OX3 7BN, United Kingdom

## Abstract

Members of four cell guidance molecule families (the netrins, slits, ephrins and semaphorins) interact with their cognate cell surface receptors to guide cells during development and maintain tissue homeostasis. Integrated structure and cell-based analyses are providing insight into the mechanisms by which these signalling systems can deliver myriad outcomes that require exquisite accuracy in timing and location. Here we review recent advances in our understanding of the roles of oligomeric states, auto-inhibition, signalling assembly size and composition in cell guidance cue function.

## Highlights

• Characterisation of a monomeric semaphorin.
• The auto-inhibition and activation mechanism of Robo ectodomain.
• Structure-guided modulation of EphA2 clustering.
• Insight into Netrin-1 complex assembly.

**Current Opinion in Structural Biology** 2020, **61**:79–85

This review comes from a themed issue on **Macromolecular assemblies**

Edited by **Xiaodong Zhang** and **Tom L Blundell**

For a complete overview see the Issue and the Editorial

Available online 17th December 2019

**https://doi.org/10.1016/j.sbi.2019.11.007**

0959-440X/© 2019 The Authors. Published by Elsevier Ltd. This is an open access article under the CC BY license (http://creativecommons.org/licenses/by/4.0/).

## Introduction

During the development of a multicellular organism the members of a few distinctive families of extracellular proteins must interact with their cognate cell surface receptors to guide cells to their correct location. Cell guidance functions continue to be essential throughout the life of the organism, be it a fly or a human, to maintain tissue homeostasis. Although ‘axon guidance’ molecules were first characterised by their role in the development of the nervous system these secreted or cell surface attached proteins are now known to perform cell guidance functions in a broad range of tissues [1]. There are four classic cell guidance molecule families: the netrins, slits, ephrins and semaphorins [2]. Various family members must work in unison to provide exquisitely detailed instructions in an appropriately timely and location-dependent manner. It has become clear that this orchestration requires multiple mechanisms to modulate both the activation and outcome of signalling through interactions occurring in the extracellular space and plasma membrane. Interestingly, evidence is emerging of direct cross-talk between the cell guidance systems at the cell surface, for example, a semaphorin receptor (PlexinA1) has been found to mediate some slit axon guidance functions [3]. However, even within one cell guidance system there are also now ample data pointing to there being multiple levels of control over the signalling output of the ligand–receptor interactions. In particular, co-receptors have been implicated in context-dependent switching of cell guidance signalling, for example, the outcome of semaphorin Sema3E binding to its receptor PlexinD1 has been reported to switch from repulsive to attractive signalling in the presence of the co-receptor neuropilin [4]. Integrated structural and cellular studies have started to provide us with some insight into the molecular mechanisms that act at the cell surface to determine divergent signalling outputs. We discuss here examples of recent advances in our understanding of how ligand state alongside receptor auto-inhibition, cluster size and composition act to control timely, location-dependent signalling functions.

## Semaphorins and plexins: adding to the ligand repertoire by varying oligomeric state

**Figure 1:**
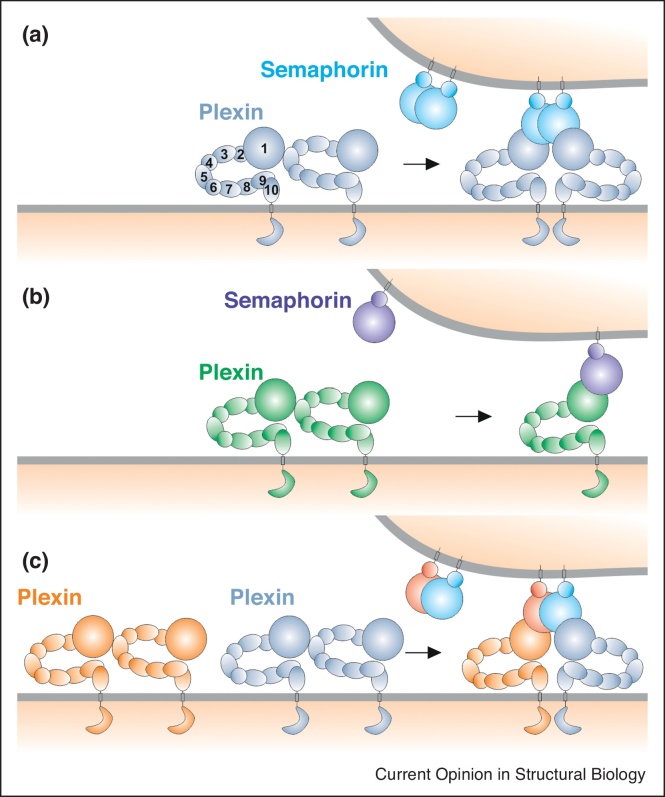
Diversity in semaphorin oligomeric state. **(a)** The structural model for semaphorin-plexin interaction. The plexin ring-like ectodomain is implicated in autoinhibition, mediating ‘head-to-stalk’ interactions between receptors on the same cell surface. Binding of semaphorin homodimer to plexin brings two plexins in close proximity which is a prerequisite for plexin signalling. **(b)** Monomeric semaphorins potentially function as antagonists as the engagement of monomeric semaphorin with the ring-like ectodomain of plexin does not lead to plexin dimerization. **(c)** Semaphorin heterodimer could bring together different members of the same plexin class, providing cross-talk between receptors that can serve as an additional level of fine-tuning of cell signalling.

Cell guidance cue receptors have diverse cytoplasmic regions, but all are type 1 single membrane spanning receptors [2]. Signalling results from changes in the oligomeric state of the receptor and the archetypal trigger for signalling is dimerisation of receptors resulting from ligand binding. The semaphorin-plexin system has been thought to exemplify this model, with a homodimeric semaphorin ligand serving to crosslink two plexin receptors [5, 6, 7]; however, recent results have revealed a previously unappreciated diversity in semaphorin oligomeric states (Figure 1).

The crystal structure of the class 1 semaphorin, *Drosophila* Sema1b, has provided the first example of a monomeric semaphorin [8^••^]. Structures of three other fly semaphorins, Sema1a, Sema2a and Sema2b, show the standard, two subunit, architecture, but with the addition of an inter-subunit disulphide bond that stabilises the dimer. Sema1b lacks the disulphide bond and various assays indicate it has no propensity to dimerise, either *in vitro* as a secreted molecule, or in its native cell surface attached form. This unexpected finding suggests that Sema1b in invertebrates, and potentially the related Sema6s in vertebrates, may mediate hereto uncharacterised biological functions as antagonists of semaphorin-plexin signalling. This study also throws a spotlight onto the role of disulphide bonds in semaphorin dimerisation. Homology modelling suggests that, similar to the fly semaphorins, the vertebrate Sema4C and Sema5s likely contain an inter-subunit disulphide bond between their N-terminal sema domains. Other semaphorins, most notably the members of the class 3 secreted semaphorins have previously been shown to have an inter-subunit disulphide located in their C-terminal tail regions. The dimerisation interface formed by the semaphorin sema domain is highly conserved within, but not between, semaphorin classes consistent with the notion that semaphorins of the same class, expressed in the same cell, may heterodimerise. Heterodimerisation was indeed demonstrated to occur for the secreted Sema2s and the Sema3s as exemplified by Sema2a/2b and Sema3A/3C heterodimers [8^••^]. This is an intriguing result because these hybrid ligands blend the properties of the two different semaphorin subunits potentially providing an additional level of fine-tuning in the semaphorin signalling repertoire. Heterodimerization has previously been shown to occur *in vitro* for members of the Nerve Growth Factor (NGF) family of cytokines [9], prompting the proposal that such hybrid ligands could display reduced but more promiscuous activity than observed for homodimers. Although heterodimers of the NGF family have not been reported *in vivo* examples of physiological heterodimers abound within the bone morphogenetic protein family [10].

## Slits and Robos: ectodomain auto-inhibition versus activation

If receptor oligomerisation is sufficient to trigger signalling the interactions of cell surface receptors in the absence of ligand must be controlled to prevent spontaneous activation. This principle has been demonstrated for the plexin receptor system [11]. Most recently a mechanism for autoinhibition has been revealed for Robo receptors. In the following we compare and contrast what we have learnt to date for these two systems.

**Figure 2:**
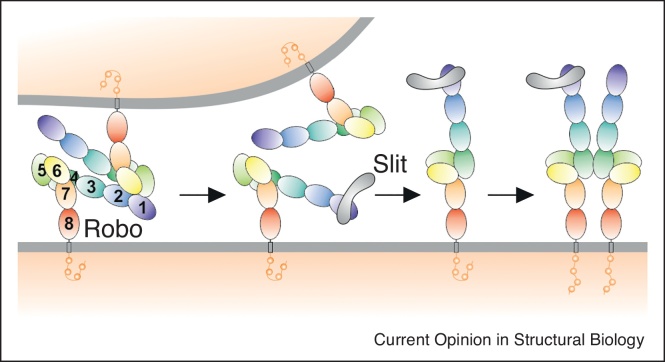
The mechanism for Robo autoinhibition and Slit-induced activation. Interaction of Robo receptors *in trans* with Robo receptors on opposing cells imposes autoinhibition. Slit binding potentially breaks the *trans* interaction leading to a conformational change that is followed by dimerization through domain D4 and signalling (adapted from Ref. [15^••^]).

The standard ectodomain in members of the Robo family of receptors contains five immunoglobulin (Ig)-like and three Fibronectin type III (FNIII) domains. A single span transmembrane region links this substantial extracellular region to an unstructured cytosolic region that provides interaction sites for intracellular effectors. Piecemeal structural dissection of the Robo ectodomain succeeded in mapping out the interaction of the N-terminal Ig-like domain with the ligand Slit [12] as well as heparan sulphate proteoglycan (HSPG) co-receptors [13]. Recent structural studies of segments of the ectodomain containing Ig-like domain 4 have also revealed its propensity to homodimerise [14^•^,15^••^]. However, insight into the mechanism by which Slit binding activates Robo has required determination of the full length ectodomain structure. A low resolution negative electron microscopy-based reconstruction, in combination with crystal structures of various segments, first provided a tantalising view of a tetrameric assembly comprising a ‘head-to-head’ arrangement of Robo1 dimers [16^•^]. In a major advance for the field, Opatowsky and colleagues have recently reported the crystal structure of the full length Robo2 ectodomain at 3.6 Å resolution [15^••^]. As surmised from the low resolution Robo1 structure, the ten domain ectodomain adopts a distinctive hairpin type structure (Figure 2). Importantly, the high resolution crystal structure reveals that the surface of domain 4 previously identified as a homodimerisation site interacts with domain 7 consistent with the hairpin being an auto-inhibited state. Furthermore, inspection of the arrangement of molecules in the crystal lattice shows two copies of the hair pin structure making a head-to-head interaction suggesting that a domain 5-mediated interaction between Robo receptors on opposing cells (i.e. *in trans*) could stabilise the auto-inhibited state. This is an exciting observation because it accords with functional studies reporting trans interactions that inhibit Robo function *in vivo* in both flies and mammals [17,18]. Conversely, functional studies have also suggested that dimerization of Robo receptors on the same cell surface (*cis* interaction) is required for activation of signalling [19]. In a beautiful demonstration of the power of integrated structural studies the detailed insights into domain 4 and domain 5 interaction interfaces were used to guide the design of cellular and *in vivo* experiments. The resulting model suggests that Slit binding acts to release the Robo ectodomain from its auto-inhibited state, the receptor can then drive its own *cis* homodimerisation to trigger signalling.

The somewhat indirect role for ligand binding in Robo activation is reminiscent of the mechanism of action of ligand binding in triggering signalling of epidermal growth factor receptor (EGFR) family members [20]. More commonly single membrane spanning receptors are activated by the ligand directly contributing to receptor dimer, or higher order, oligomerisation. However, even for examples such as the semaphorin-plexin system, in which the dimeric semaphorin serves to crosslink two plexin receptors, auto-inhibition mechanisms have been shown to be essential. One common theme that has emerged is the active role played by the large, multidomain ectodomains of receptors in the modulation of their signalling. The Robo ectodomain comprises eight domains, the Plexin ectodomain typically contains ten domains. Structural studies have now revealed that Robo and Plexin ectodomains are not linear beads on string arrangements, but rather can adopt hairpin or ring-like conformations conferring distinct functional properties. The Robo hairpin provides a mechanism for auto-inhibition within a single receptor, in contrast the plexin ectodomain can mediate ‘head-to-stalk’ interactions between receptors that prevent them signalling pre-semaphorin binding [11] (Figure 1).

## Ephrins and Ephs: cluster size and signal outcome

Signal complex composition can vary in terms of cluster size and composition. Both mechanisms have been found to play a role in the functional outcome for cell guidance cue signalling. In this section we discuss advances in our understanding of the ephrin-Eph cluster-based signalling system.

Ephrins are cell surface attached ligands that signal through members of the Eph family of receptor tyrosine kinases. The role of dimerisation in receptor tyrosine kinase signalling is well documented [20]; however, higher order oligomerisation is required to trigger Eph signalling [2]. The required size of the cluster, and consequent signal output, appears to vary between Ephs. Visualisation of EphB2 receptors in living cells indicated that for this receptor activation occurs through trimers and tetramers, with dimerization failing to initiate the cell collapse response characteristic of repulsive signalling [21]. More recently advanced light microscopy analyses have been applied to dissect the spatiotemporal characteristics of EphB2 activation [22^•^]. The results suggest a model in which the receptor is initially activated by the formation of 6-mer to 8-mer oligomers, higher order interactions between these oligomers can then generate larger clusters that dampen signalling. In an approach using supported lipid bilayers for ligand presentation, Grove and colleagues imposed physical restrictions (so called spatial mutation) on the movement and assembly of EphB4 - ephrinB2 signalling clusters by the introduction of nano-fabricated barriers [23^•^]. Intriguingly, the experiment showed that the ability of EphB4-ephrin-B2 signalling to induce neuronal differentiation can be modulated by spatiomechanical effects. At the level of molecular structure a series of crystallographic analyses, integrated with cellular studies, have revealed that a combination of ligand-receptor and *cis* receptor-receptor ectodomain interactions confers distinctive clustering capabilities, and signalling outputs, on Eph family members [24, 25, 26, 27]. In addition to the kinase domain the Eph cytoplasmic region contains a SAM domain. The specific pairwise interactions of Eph and effector SAM domains have been recently highlighted as a source of diversity in signalling outcome [28^•^].

**Figure 3:**
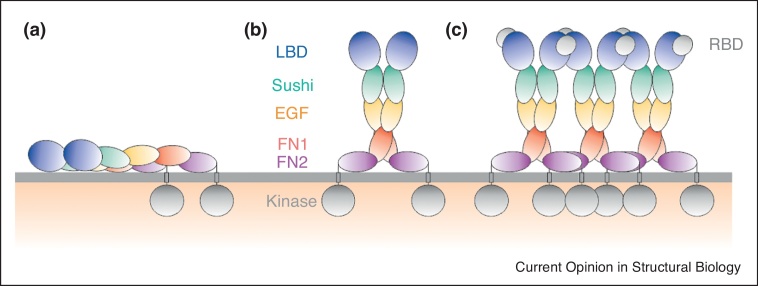
Structural model of EphA2 receptors at the membrane. **(a)** Unliganded EphA2 dimer with two ectodomains lying parallel on the membrane with the two separated kinase domains. **(b)** The kinase domains of the dimer are still separated in the liganded dimer arrangement. **(c)** Interactions between adjacent dimers within a higher order receptor cluster allow autophosphorylation of kinase domains (adapted from Ref. [31^•^]). Ligand-binding domain (LBD), epidermal growth factor like (EGF), fibronectin type III (FN1 and FN2), receptor-binding domain (RBD).

The interactions of receptor and plasma membrane are increasingly recognised as contributing to the mechanism of action of receptor tyrosine kinases (for example [29]). Molecular dynamics-based approaches have been used to gain insight into the role of ectodomain-membrane interactions in Eph clustering [30]. More recently these studies have been developed to include the potential contributions of membrane-mediated interactions with the juxtamembrane and kinase regions of the receptor [31^•^]. In combination with the previous structural and cellular studies, these *in silico* analyses suggest a model in which the balance of receptor–receptor, receptor–lipid and receptor–ligand interactions controls the switch from inactive dimer-based receptor states to signalling clusters (Figure 3). Such molecular-level insights into mechanism may be of direct utility for biomedical applications. Aberrant EphA2 signalling is implicated in a number of pathologies, including cancer, and strategies to modulate EphA2 clustering are under active investigation. In an exciting step forward for the field, Pasquale *et al*.–Gomez-Soler *et al* have used structure-guided design to engineer peptides with nanomolar potency that can differentially modulate EphA2 clustering [32^••^].

## Netrin and DCC: modulating signal by complex composition

There are numerous reports of cell guidance signalling systems switching their functional output according to complex composition, however, we still have very limited insight into the mechanisms that control these effects. Here we survey the state of our knowledge for two cell guidance systems where some structural information on the effect of additional components is available.

The co-receptor neuropilin is essential for secreted class 3 semaphorin signalling through class A plexins. Structural analysis of a Sema3A–PlxnA2–Neuropilin1 complex has indicated that the N-terminal domain of the neuropilin ectodomain serves as molecular glue, holding together an otherwise unstable semaphoring-plexin complex by the addition of cross-bracing semaphoring-neuropilin and neuropilin–plexin interactions [33]. However, the role of neuropilin binding is clearly more complex than a simple stabilizer of the canonical semaphoring-plexin interaction as functional studies of the Sema3E-PlexinD system indicate that in the presence of the neuropilin the signal outcome switches functionality from repulsion to adhesion [4]. Apart for the limited understanding of neuropilin, there is a dearth of mechanistic insight for a rapidly expanding range of reported co-receptors that include regulatory receptors of the immune system and cell adhesion molecules [34,35].

**Figure 4:**
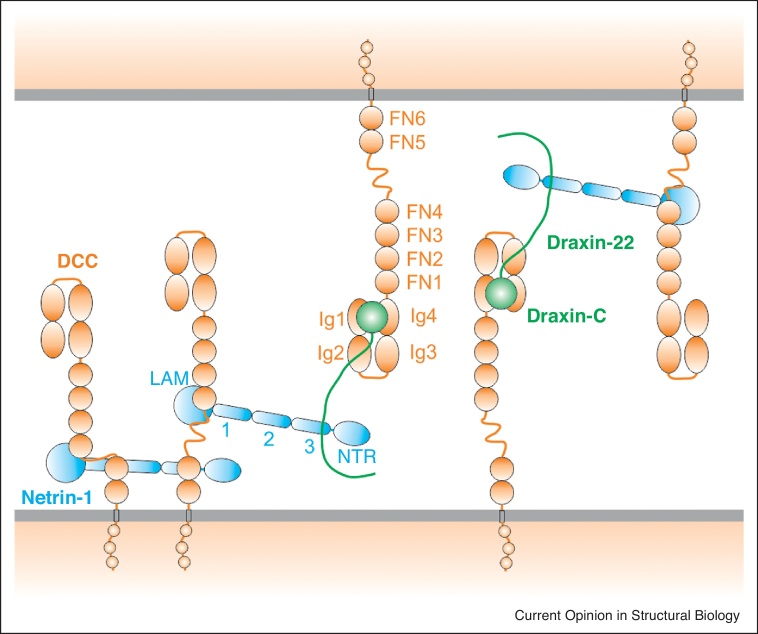
Structural model for Netrin1-Draxin-mediated adhesion between axons through DCC. Multiple interaction sites provided by the multi-domain architecture of the DCC ectodomain promote formation of an adhesive array between axons (adapted from Ref. [39^•^]).

The cell guidance cue Netrin-1 and its receptors DCC and UNC5 exemplify a signalling system which can have diametrically opposing outputs depending on the composition of the signalling complex. The interaction of Netrin-1 with DCC results in an attractive response, conversely the presence of UNC5 results in a repulsive guidance signal [36]. The interaction of Netrin-1 and UNC5 still awaits structural characterisation. The DCC ectodomain comprises four Ig-like domains followed by six FN3 domains. Molecular level models of signalling assemblies have been derived from crystal structures of Netrin-1 in complex with FN3-containing segments [37,38]. Recent structural studies have probed the role of a second guidance cue, Draxin, detailing its interactions with Netrin-1 and with the N-terminal four Ig-like domains of DCC [39^•^]. These results, when combined with the previous studies on Netrin-1-DCC complexes, suggested a model in which Draxin and Netrin-1 can jointly contribute to DCC mediated-adhesion between axons (Figure 4).

## Concluding remarks

In the above sections we have discussed some of the latest insights into the roles of oligomeric states, auto-inhibition, signalling assembly size and composition in cell guidance cue function. Signalling assembly composition is likely central to the myriad functional outcomes triggered by cell guidance cues and there is clearly much to explore. Cell guidance receptors have been shown to form supercomplexes with members of very different receptor families [40] and interactions with proteoglycans offer additional modes of regulation [41]. We have focused this review on the interactions at the cell surface that trigger or modulate signalling. It is clear that much also awaits discovery regarding the mechanisms activated within the cell, for example, structural studies are beginning to probe the connection between plexin signalling and receptor endocytosis [42^••^].

Cell guidance signalling systems are exquisitely tuned to control the development of complex tissues and circuitry during embryogenesis, and to maintain these structures in the adult organism. Recent studies of human class 3 semaphorin variants point to the importance of this fine tuning and underscore how much there is yet to understand [43^•^]. A mechanistic understanding of cell guidance signalling ultimately requires the integration of structure and cell-based analyses with *in vivo* studies. Indeed, the challenge for the field is to design studies of sufficient range, in space and time, to provide molecular level insight into the biology.

## Conflict of interest statement

Nothing declared.

## References and recommended reading

Papers of particular interest, published within the period of review, have been highlighted as:

• of special interest
•• of outstanding interest
